# Active water management brings possibility restoration to degraded lakes in dryland regions: a case study of Lop Nur, China

**DOI:** 10.1038/s41598-022-23462-9

**Published:** 2022-11-03

**Authors:** Shanlong Lu, Yong Wang, Jinfeng Zhou, Alice C. Hughes, Mingyang Li, Cong Du, Xiaohong Yang, Yutong Xiong, Feng Zi, Wenzhong Wang, Zhaoxian Zheng, Chun Fang, Shunli Yu

**Affiliations:** 1 International Research Center of Big Data for Sustainable Development Goals, Aerospace Information Research Institute, Chinese Academy of Sciences, Beijing, 100101 China; 2 University of Chinese Academy of Sciences, Beijing, 100049 China; 3 China Biodiversity Conservation and Green Development Foundation, Beijing, 100089 China; 4 Hunan University of Science and Technology, Xiangtan, 411201 China; 5 Centre for Integrative Conservation, Xishuangbanna Tropical Botanical Garden, Chinese Academy of Sciences, Xishuangbanna, 666100 China; 6 Institute of Hydrogeology and Environmental Geology, CAGS, Shijiazhuang, 050061 China; 7 Institute of Botany, Chinese Academy of Sciences, Beijing, 100093 China

**Keywords:** Climate change, Hydrology, Hydrology

## Abstract

Protecting and restoring the degraded arid lakes are globally urgent issues. We document a potential recovery of the dried salt-lake, Lop Nur called "the Sea of Death" which is located at the terminus of the largest inland basin in China, the Tarim River Basin. The changes and relationship of surface water with climate parameters and groundwater in the basin over the last 30 years are analyzed, by using satellite remote sensing and land data assimilation products. We find that with increased surface water in the basin, the groundwater level in Lop Nur began to show an obvious positive response in 2015; and the rate of decline of the groundwater level is slowing down. We argue that after a balance is achieved between regional groundwater recharge and evapotranspiration, the Lop Nur ecosystem will gradually recover. This study shows an encouraging case for the protection and restoration of degraded lakes in dryland regions around the world.

## Introduction

In recent decades, under the influence of climate change and human activities, the shrinkage and degradation of lakes is common in global arid areas, such as the disappearance of the Aral Sea in Central Asia^1^, the shrinking and degraded Daihai Lake and Huangqihai Lake on the Inner Mongolian Plateau^2,3^, the critical state of the Great Salt Lake of the United States^4^, and the drying up Lake Urmia in Northern Iran^5^, etc. In order to protect and restore these vulnerable and unique ecosystems and achieve a sustainable future for the life that depends on them, intense conservation efforts and recovery actions are urgently required^6–8^. Here we document an arid lake in potential recovery, Lop Nur, called “the Sea of Death,” which has experienced complete drying but is now on the road to recovery.

**Figure 1:**
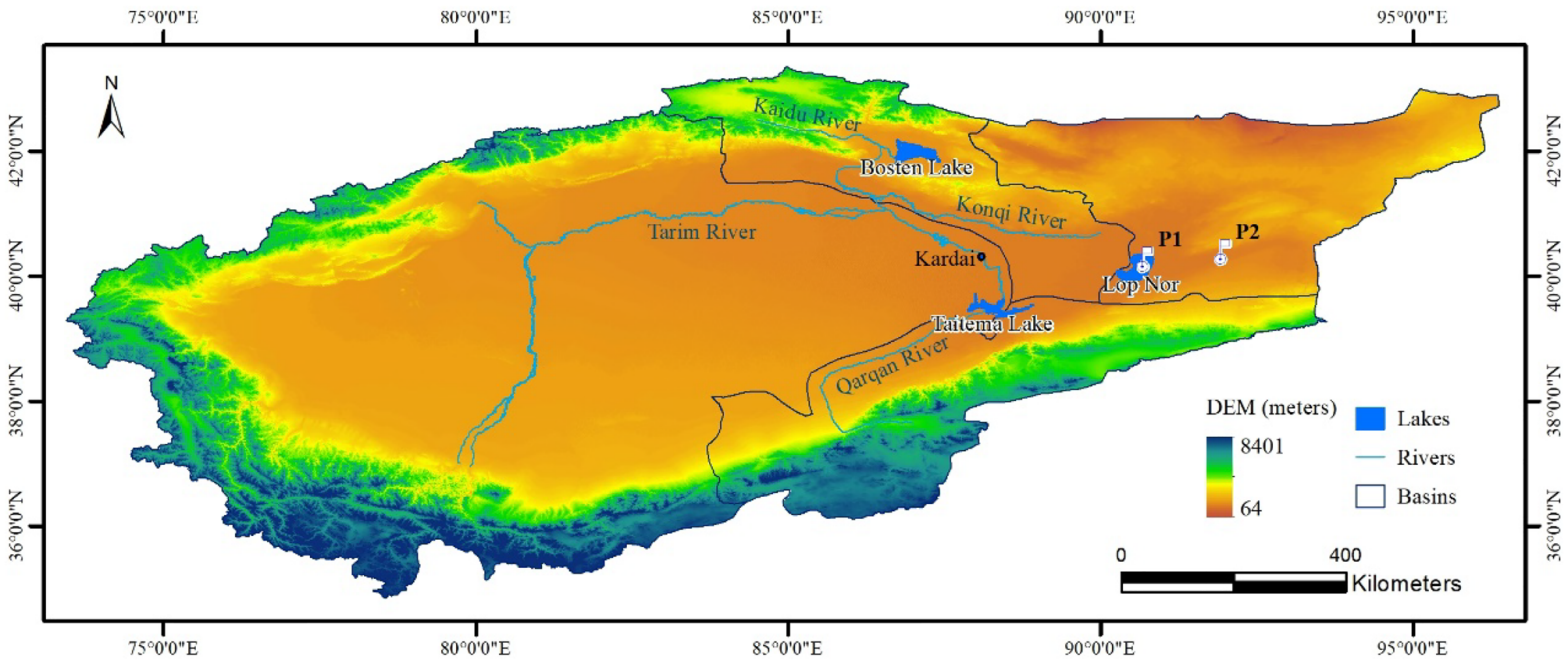
Digital terrain and surface water system of the Tarim River Basin (P1, P2 are groundwater field observation locations). This figure was generated by ArcGIS v10.7 software (Environmental Systems Research Institute, Inc., USA, URL: http://www.esri.com/).

Lop Nur is a dried salt-lake basin located between the Taklamakan and Kumtag Deserts in the southeastern portion of the Xinjiang Uygur Autonomous Region of China (Fig. 1), at the terminus of the Tarim River Basin, which is the largest inland basin in China. It is one of the most mysterious places on earth because of its role in the ancient Silk Road civilization^9^, the controversy over the disappearance of the Great Lakes^10^, nuclear testing^9^, and the special shape of the "Great Ear" in satellite views^11^.

According to historical data, around 220 AD, Lop Nur was the largest lake in China, with an area of 5350 square kilometers^12^, and carbon data shows that the lake survived over 20,000 years before starting to shrink around 400 years ago following a period when it supported a thriving community of humans and native ecosystems^13^. By 1931 the lake measured 1900 square kilometers^14^. After large-scale upstream regional agricultural development in 1950, the water surface of Lop Nur gradually decreased until the lake dried up entirely in 1962, which was rediscovered by the first earth resources satellite of the United States^15^.

Lop Nur dried up because of decreased water flow from its two main tributaries, the Tarim River and Konqi River, caused by unsustainable water development and water utilization activities such as the expansion of cultivated land, overgrazing, and construction of water conservancy projects^16^. The decreased water from the upper reaches also led to the rapid deterioration of the two rivers, including river closure, the rapid decline of groundwater levels, degradation of native *Populus euphratica* forests, aggravation of desertification, damage to biodiversity (including the last wild herd of Bactrian camels), and a suite of other consequences^17^.

Development and excessive utilization of water resources in the region has aroused widespread concern among academia and the government^18^. Restoration and water replenishment of the Tarim and Konqi Rivers began in 2000 and 2015, respectively, and has enabled the recovery of the lakes and wetland ecosystems in the lower river reaches^19,20^, bringing hope for the resurrection of Lop Nur, the terminal lake of the two rivers. Furthermore, over the last 50 years, regional climate warming and wetting (increases in temperature and precipitation)^21,22^ have increased the water resources of the Tarim River Basin^23^. This trend is projected to continue to at least the middle of the twenty-first century^24^, which also increases the possibility of the rebirth of Lop Nur. Because this arid lake dried up from the double negative effects of climate change and human activities, documenting its recovery under the double positive effects of climate change and human activities and inspires hope for the restoration of a place with an important role in human history.

In this study, we analyze the causes of the change in the regional surface water area and its impact on the groundwater level in Lop Nur. We then assess the possibility of future recovery of Lop Nur, based on an analysis of the change in the surface water area and meteorological elements of the Tarim River Basin, the groundwater level in Lop Nur, and the surface water in different tributary rivers.

### Surface water changes in the Tarim River Basin

**Figure 2:**
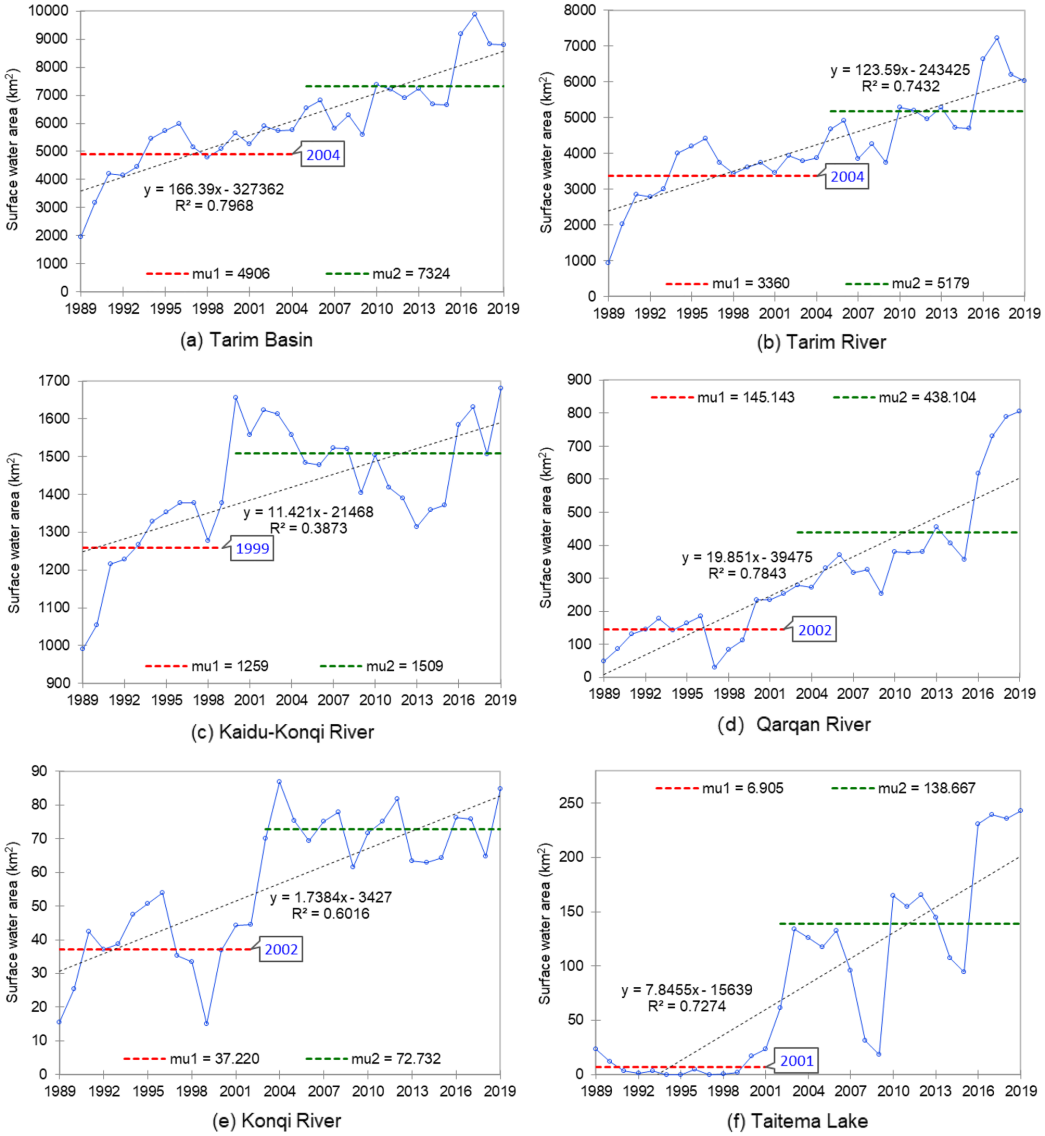
Variation trend and tipping point change of surface water area in the Tarim River Basin and its tributaries from 1989 to 2019.

In the last 30 years, the surface water area of the Tarim River Basin and all tributary rivers has increased significantly. Among the rivers, the Qarqan River has the highest rate of increase, followed by the Tarim River and the Kaidu-Konqi River (Table 1). The results of Pettitt’s test show that the surface water area of the Kaidu–Konqi River and Qarqan River experienced abrupt changes in 1999 and 2002, respectively (Fig. 2c, Fig. 2d, Table 2), while that of the Tarim River and the whole basin did so in 2004 (Fig. 2a, Fig. 2b, Table 2). In terms of spatial distribution, the downstream surface water area of these three tributaries shows an obvious increasing trend: the surface water area of the Konqi River downstream of Bosten Lake and the Kaidu River also shows a significant increase, with an abrupt change in 2002 (Fig. 2e, Table 2); the surface water area of Taitema Lake, the terminal lake of the Qarqan and Tarim Rivers in recent decades, shows a significant increase, with an abrupt change in 2001 (Fig. 2f, Table 2). The temporal and spatial variation of surface water in the basin may have an impact on the variables of the regional groundwater system.

**Table 1:** Mann–Kendall (M–K) trend test results of surface water area in the Tarim River Basin and its tributary rivers and terminal lake.

	Kendall's tau	*p* value (two-tailed)	alpha
Tarim river	0.686	< 0.0001	0.05
Tarim basin	0.751	< 0.0001	0.05
Kaidu-Konqi river	0.419	0.001	0.05
Qarqan river	0.806	< 0.0001	0.05
Konqi river	0.548	< 0.0001	0.05
Taitema lake	0.617	< 0.0001	0.05

**Table 2:** Pettitt's test results of surface water area in the Tarim River Basin and its tributary rivers and terminal lake.

	Change year	*p* value (two-tailed)	alpha
Tarim basin	2004	< 0.0001	0.05
Tarim river	2004	0.000	0.05
Kaidu-Konqi river	1999	< 0.0001	0.05
Qarqan river	2002	< 0.0001	0.05
Konqi river	2002	< 0.0001	0.05
Taitema lake	2001	< 0.0001	0.05

### Groundwater changes in the Lop Nur region

Since 1962 Lop Nur has been dry, which experiences a severely dry climate with rainfall less than 50 mm and a high evaporation rate of 2728 mm annually^25^. The land cover types are salt crust, Gobi, and sand. Because there has been no surface runoff, change in soil water and surface water can be ignored on an annual scale. Therefore, the change in total land water storage retrieved by the GRACE satellites can be used to reflect the change in groundwater storage:

$$ \Delta GW = \Delta TWS - \Delta SM - \Delta SWE $$

where, $\Delta GW$ is groundwater change, $\Delta TWS$ is the change in total water storage, $\Delta SM$ and $\Delta SWE$ are the change in soil water and surface water, respectively, with value of zero.

**Figure 3:**
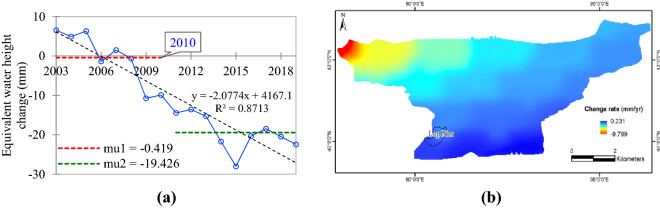
Variation trend and tipping point change (a) and spatial change rate (b) of equivalent water height of groundwater in the Lop Nur region from 2003 to 2019. The right figure was generated by ArcGIS v10.7 software (Environmental Systems Research Institute, Inc., USA, URL: http://www.esri.com/).

Based on the trend analysis results of the Mascons GRACE product (equivalent water height data) obtained by the GRACE satellites^26^ in the Lop Nur region from 2003 to 2019, the groundwater level in the region has shown a significant decrease, but the decrease was detected slowed down since 2010 and an obvious reversal trend occurred in 2015 (Fig. 3a). Similar results are found in the lower reaches of Kaidu-Konqi River basin based on observation and remote sensing detection^27^.

The trend in equivalent water height spatial change (changes in each pixel) shows that the groundwater level in the Lop Nur region (especially the southern and eastern areas) has exhibited a recovery trend (Fig. 3b).

### Impacts of climate change on surface water

**Figure 4:**
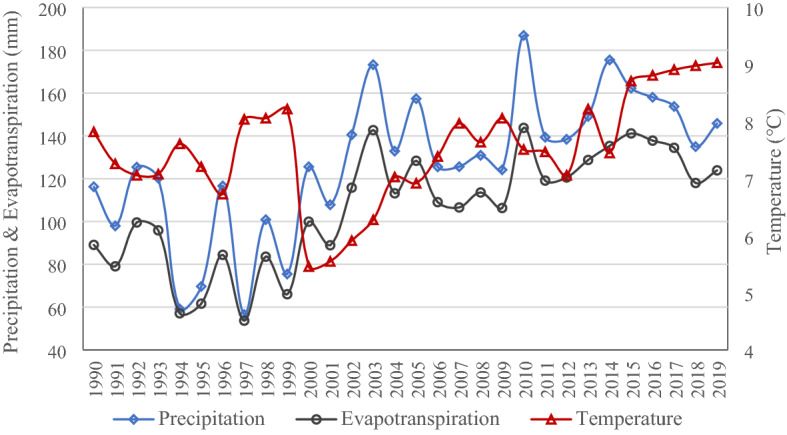
Change trends of annual precipitation, evapotranspiration, and average temperature from 1989 to 2019.

In the Tarim River Basin, the change in surface water area is affected mainly by changes in precipitation, glacial meltwater, and evapotranspiration. Over the last 30 years, annual precipitation, annual average temperature, and annual evapotranspiration in the basin have increased significantly, but the annual average temperature has increased relatively slowly (Fig. 4). The results of Pettitt’s test show that the annual precipitation and evapotranspiration experienced an abrupt change in 2001, while annual average temperature did so in 2006. These changes in the tipping point of annual precipitation and evapotranspiration are closely connected with that of changes in the surface water area of the basin and tributary systems (Table 3, Fig. 2), indicating their tight impact on increasing the change in surface water area in the basin.

**Table 3:** Pettitt's test of the annual precipitation, evapotranspiration, and average temperature.

Pettitt's test	Precipitation	Evapotranspiration	Temperature
Change year	2001	2001	2006
*p* value (two-tailed)	< 0.0001	< 0.0001	0.005
alpha	0.05	0.05	0.05

Pearson’s correlation test results show significant correlation characteristics between the surface water area of each tributary river and the annual precipitation, evapotranspiration, and average temperature in the whole basin. The Pearson’s correlation coefficient of annual evapotranspiration is the highest, followed by annual precipitation and annual average temperature. The annual precipitation and evapotranspiration in the basin are positively correlated with the surface water area of each river (Table 4). Possible reasons are as follows: 1) the expansion of surface water area provides more underlying surface prone to evaporation^28^; 2) the increased temperature provides sufficient heat for evaporation. The evapotranspiration in the basin increased with the increased surface water area synchronously, which means that the increase in precipitation in the region and the increase in glacial meltwater caused by the increasing temperature are larger than the increased evapotranspiration and so are the main reasons for the increase in surface water area in the basin.

**Table 4:** Pearson’s correlation test results between meteorological elements in the Tarim River Basin and surface water area of each tributary river.

Meteorological elements in the basin	Correlation analysis parameters	Surface water area of each river		
		Qarqan River	Kaidu-Konqi River	Tarim River
Annual average temperature	Correlation coefficient	0.509**	− 0.054	0.491**
	Significance (bilateral)	0.003	0.772	0.005
	Number of samples	31	31	31
Annual precipitation	Correlation coefficient	0.613**	0.417*	0.490**
	Significance (bilateral)	0.000	0.019	0.005
	Number of samples	31	31	31
Annual evapotranspiration	Correlation coefficient	0.696**	0.474**	0.577**
	Significance (bilateral)	0.000	0.007	0.001
	Number of samples	31	31	31

*Significance level with alpha = 0.05, **Significance level with alpha = 0.01.

Based on the glacier evolution scheme and precipitation-runoff model results, the contribution of precipitation (including rainfall and snowmelt) and glacier melt to the surface runoff was 71.8% and 28.2% respectively on average over the period from 1971 to 2010 in a headwater basin of the Tarim River^29^. Another estimation shown that the average glacier melt contribution to total runoff is 30–37%^30^. According to the latest precipitation scenario simulation results, the future (2021 ~ 2100) precipitation of the Tarim River Basin will increase significantly relative to the base period (1961 ~ 2005)^31^. The melting water volume of glaciers will also increase with the glacier shrinkage until about 2050^30^. Consequently, they will have a continuous positive contribution to the regional surface water before the reverse of the climate change trend.

### Impacts of human activities on surface water

**Figure 5:**
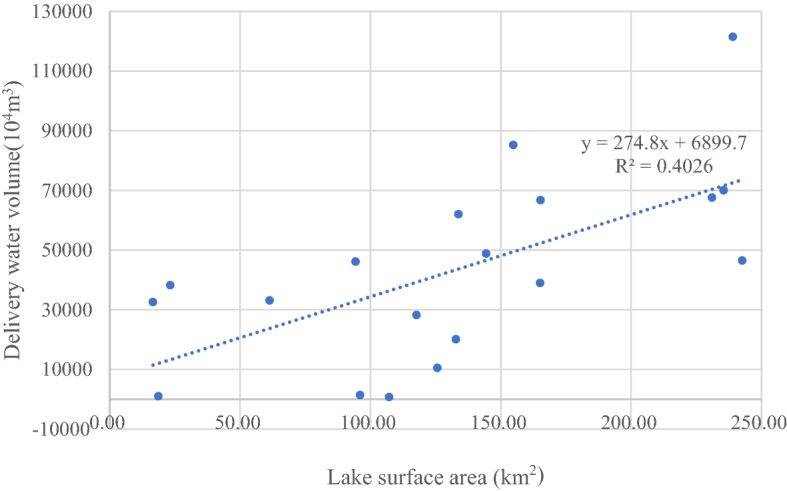
Linear correlation between surface water area of Taitema Lake and the total annual volume of ecological water delivery of Tarim River.

In 2001, the Chinese government launched a comprehensive management and restoration project in the Tarim River Basin^32^, aiming to resurrect the lake and restore riparian and surrounding ecosystems across the basin. Water was delivered from upstream to the lower reaches of the Tarim River 21 times for ecological recovery, accumulating 8.45 billion cubic meters^33^. Since these 20 years of ecological supplementation measures, the surface water distribution in the region has changed significantly. The surface water area of Taitema Lake has also continued to increase since 2001 (Fig. 2f). This change is directly related to the ecological water delivery of the upper Tarim River. The monitoring statistics of the river section affected by the ecological water delivery show that during the ecological water delivery period from April to November 2001, the water head of the Tarim River reached the Taitema Lake for the first time after 30 years cutoff (Table 5). Furthermore, a linear relationship between the surface water area of Taitema Lake and the annual total volume of the delivered water was found (Fig. 5), which directly reflects the impact of ecological water delivery on water recovery of Taitema Lake. With this process the ecosystem in the lower reaches of the Tarim River has gradually recovered, the native vegetation area in the lower reaches of the Tarim River has increased^20^.

**Table 5:** Ecological water delivery number, data, volume, and water head reached area of the Tarim River (Wang and Tursun, 2020).

Number of ecological water delivery	Date	Volume(10^4^m^3^)	Water head reached area
1st	2000–05 ~ 2000–07	9923	Kardai
2nd	2000–11 ~ 2001–02	22,655	Kardai
Phase 1 of the 3rd	2001–04 ~ 2001–07	18,433	Taitma lake
Phase 2 of the 3rd	2001–09 ~ 2001–11	19,790	Taitma lake
4th	2002–07 ~ 2002–11	33,129	Taitma lake
Phase 1 of the 5th	2003–03 ~ 2003–07	34,028	Taitma lake
Phase 2 of the 5th	2003–08 ~ 2003–11	27,997	Taitma lake
6th	2004–04 ~ 2004–06	10,527	Taitma lake
Phase 1 of the 7th	2005–04 ~ 2005–06	5236	Taitma lake
Phase 2 of the 7th	2005–08 ~ 2005–11	22,997	Taitma lake
8th	2006–09 ~ 2006–11	20,098	Taitma lake
9th	2007–09 ~ 2007–11	1411	Kardai
10th	2009–11 ~ 2009–12	1027	Kardai
11th	2010–06 ~ 2010–11	38,952	Taitma lake
12th	2011–04 ~ 2011–11	85,211	Taitma lake
13th	2012–04 ~ 2012–11	66,716	Taitma lake
14th	2013–04 ~ 2013–10	48,800	Taitma lake
15th	2014–06	727	Taitma lake
16th	2015–08 ~ 2015–11	46,128	Taitma lake
17th	2016–08 ~ 2016–10	67,611	Taitma lake
18th	2017–04 ~ 2018–01	121,461	Taitma lake
19th	2018–04 ~ 2018–11	70,006	Taitma lake
20th	2019–08 ~ 2019–12	46,482	Taitma lake
21th	2020–09 ~ 2020–11	27,934	Taitma lake
Total		847,279

Since 2015, the local government has launched an action to save the Konqi River. To achieve this, water has been diverted from the Tarim River and Bosten Lake and the middle and lower reaches of the Konqi River 5 times (once each year from 2016 to 2020), with cumulative water delivery of 1.57 billion cubic meters, which has rejuvenated hundreds of kilometers of rivers that have been cut off for many years^34^. Access to water has enabled recovery of native ecosystems, and vegetation has increased significantly as a consequence^35^.

### Influence of surface water change on groundwater level in Lop Nur

**Figure 6:**
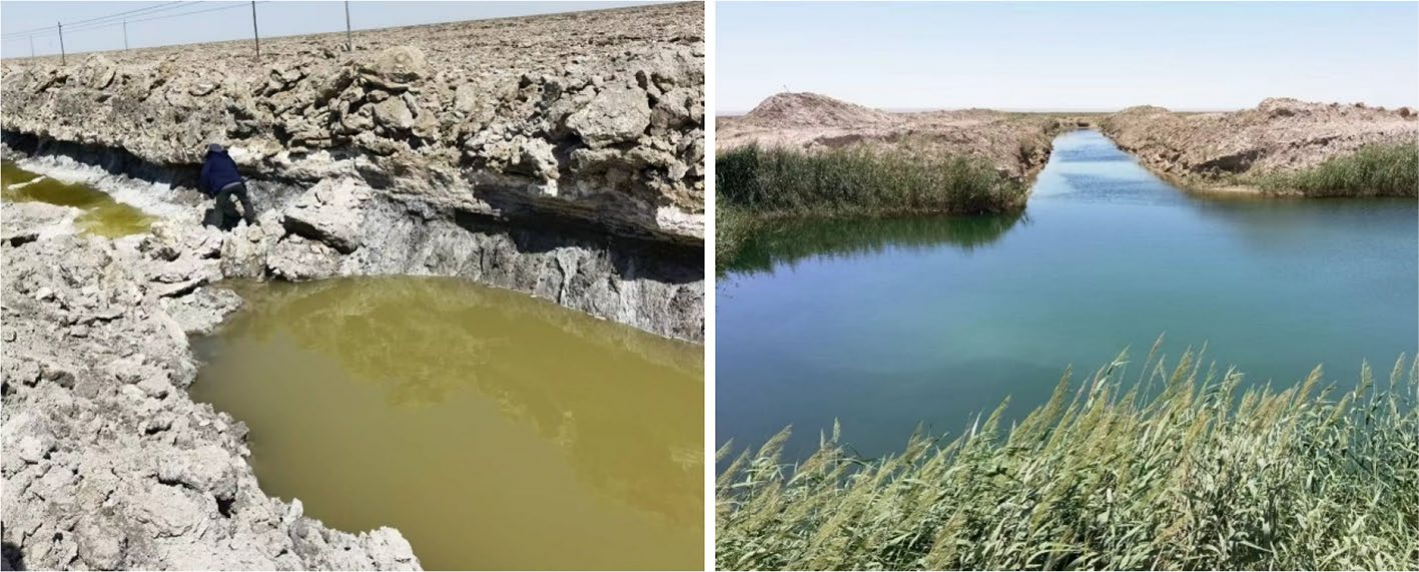
Burial depth of groundwater in central (Fig. 1, P1) and east (Fig. 1, P1) of Lop Nor region (The photo was taken by the corresponding author on July 27 & 28, 2021).

The increased surface water in each tributary river in the Tarim River Basin has raised the groundwater level in each river basin^34,36^ and has also had an impact on the groundwater level in Lop Nur. In order to characterize the influence of the three rivers on the change in the groundwater level in the Lop Nur region, the correlation between the surface water area of the three major rivers and the equivalent water height in Lop Nur before and after 2015 is analyzed respectively. The results show that there is an obvious positive correlation between the changes in surface water area of the three rivers and the equivalent water height in Lop Nur during 2015 ~ 2019, with the change in the surface water area of the Tarim River having the strongest correlation with the equivalent water height. But before 2015 (2003 ~ 2014), the surface water area of the Tarim River and Qarqan River and the equivalent water height in Lop Nur had a negative correlation (Table 6). These results mean that before 2015, the annual recharge of groundwater by the rivers upstream of Lop Nur is less than the total evapotranspiration, resulting in the gradual decrease in groundwater. After 2015, the difference between groundwater recharge and regional evapotranspiration gradually decreased, followed by the declining groundwater decrease. Although the groundwater in the area is still decreasing, it has been in a positive recovery state (Fig. 3). This process is exemplified by the restoration of groundwater level reflected by the observation data of groundwater level on both sides of the lower reaches of the Kongqi River under the influence of ecological water delivery from 2015 to 2019. During this period, the average annual groundwater level rises by 0.6 ~ 0.98m^34^. Based on the field survey results, the buried depth of groundwater in the core area (Fig. 1, P1) and the edge area (Fig. 1, P2) of Lop Nur is only about 1.5 ~ 2.0 m (Fig. 6). So it can be inferred that the groundwater level in the Lop Nur region will further recover in the future without additional changes in the regional climate trend, ecological water delivery intensity, and groundwater exploitation^37^ intensity. After a balance is achieved between the regional groundwater recharge and evapotranspiration, the Lop Nur ecosystem will gradually recover.

**Table 6:** Pearson’s correlation between equivalent water height in the Lop Nur region and the water surface area of each tributary river during two periods (2003 ~ 2014, 2015 ~ 2019).

2003 ~ 2014				
		**Tarim river**	**Qarqan river**	**Kaidu–Konqi river**
Equivalent water height in Lop Nur region	Correlation coefficient	− 0.582*	− 0.660*	0.863**
	Significance (bilateral)	0.047	0.020	0.000
	*N*	12	12	12
**2015 ~ 2019**				
Equivalent water height in Lop Nur region	Correlation coefficient	0.978**	0.787	0.729
	Significance (bilateral)	0.004	0.114	0.163
	*N*	5	5	5

*Significance level with alpha = 0.05, **Significance level with alpha = 0.01.

## Conclusions

Based on satellite remote sensing surface and groundwater datasets, through non-parametric trend analysis, abrupt change time detection analysis, and single factor correlation analysis, this paper analyzes the change in surface water area in the Tarim River Basin where Lop Nur is located, under the influence of climate change and human activities, and its impact on the change in groundwater level in Lop Nur.

1. In the last 30 years, the surface water area of the Tarim River, Qarqan River, and Kaidu-Konqi River in the Tarim River Basin has increased significantly;
2. The increased precipitation in the region is the main reason for the increase in surface water area in the basin;
3. Water delivery has a significant impact on the spatial distribution of surface water of the Tarim River and Konqi River. In 2000, ecological water delivery began in the Tarim River from upstream to the lower reaches, and the surface water area of Taitema Lake showed an abrupt change in 2001;
4. The decreasing trend of groundwater level in the Lop Nur region slowed down since 2010 and an obvious reversal trend occurred since 2015, and this change has the strongest correlation with the change in surface water area of the Tarim River;
5. Without unanticipated changes in the regional climate trend, ecological water delivery intensity, and groundwater exploitation intensity, the groundwater level in the Lop Nur region will be restored partially or completely in the future. After the regional groundwater in Lop Nur reaches a balance between gains and losses, the regional water ecosystem will gradually recover.

Lop Nur is a large lake dried up under the influence of climate change and human activities in an arid region. According to this study, it may recover under the dual influence of climate change and human governance in the future. This study has important reference value for lake ecological protection and restoration activities in global arid and semi-arid regions (especially in Central Asia adjacent to the study area) and adaptations to the impacts of climate change in the future.

## Methods

### Surface water data

The surface water data were extracted from the JRC Yearly Water Classification History (V1.3) dataset within the Google Earth Engine. This dataset was generated from millions of satellite remote sensing images of Landsat 5, 7, and 8 acquired between March 16, 1984, and December 31, 2020^37–39^. It has been widely used after openly shared due to its’ relatively high accuracy and spatio-temporal continuity^40,41^. The cross comparison with the Time series of Inland Surface Water Dataset in China (ISWDC)^42^ and the local dataset in Tarim River basin^43^ also shown very high consistency of spatial distribution pattern and temporal trends. The total water area including permanent and seasonal water area was used here. The data in 1995 were interpolated by calculating the mean value of adjacent years (1994 and 1996) due to abnormal values in the study area.

### Climate data

The precipitation, temperature, and evapotranspiration data were extracted from the hydrological model data in near real-time land surface change data simulated by global land data assimilation systems (GLADAS)^44^, including GLDAS_NOAH025_M2. 0 model data from 1989 to 1999 and GLDAS_NOAH025_M2.1 model data from 2000 to 2019, with a spatial resolution of 0.25° and a temporal resolution of 1 month. The previous research shows that GLADS hydrological model data has a high accuracy in reflecting the precipitation changes the evapotranspiration on the Tibetan plateau^45,46^ while the temperature data is generally accurate in the global scale^47^. This data set has been successfully applied to the study of the relationship between climate change, terrestrial water storage and agricultural water consumption in the Tarim River basin^48,49^. The total precipitation (rainfall and snowfall), temperature, and evapotranspiration data for 367 months in the study area for 31 years are used. The data for 5 months, from June 2002 to September 2002 and January 2016, are missing—for these years (2002 and 2016) with missing data, the mean value of adjacent years was used.

### Groundwater data

The equivalent water height data were obtained from the dataset of the GRACE-FO RL06 Mascon solutions (Version 02), which are generated by the Center for Space Research, University of Texas at Austin^50^. This dataset is better than other algorithm-generated results^26^. Yang (2021) obtained the groundwater storage change trend of Kongqi River basin based on this dataset, which is consistent with the results obtained from field observation. In this study, the monthly 0.25° resolution data from 2003 to 2019 were used. The data in 2018 were replaced by the mean value of adjacent years due to obvious data anomalies.

### Mann–Kendall (M–K) trend test

The Mann–Kendall test is a nonparametric test^51,52^. Its advantage is that a few outliers have little effect on it, and the samples do not need to follow a certain distribution. It is widely used in variable trend detection at different scales^53^. The Kendall's tau is the trend value. When $tau$>0, it indicates that the sample has an increasing trend; when $tau$ is negative, it indicates a decreasing trend. The alpha is the significance level, and *p* value is the parameter to decide reject or accept the null hypothesis.

### Pettitt’s test

Pettitt's test is an improved algorithm based on the M–K test. It can identify the point of mutation in time series data^54^. It is a common tool to detect a single unknown mutation point in a time series and is one of the common nonparametric test method^53^. The alpha is the significance level, and p-value is the parameter to decide reject or accept the null hypothesis. It is generally believed that when p-value is less than 5%, the change is significant, which means the sequence experienced a mutation at this point.

### Bivariate Pearson’s correlation test

Pearson’s correlation coefficient includes standard deviation and covariance, which are used to represent the numerical characteristics of the relationship between two variables. The closer its absolute value is to 1, the better the correlation is. The closer its absolute value is to 0, the worse the correlation is—0 means no correlation, a positive number means a positive correlation, and a negative number means no correlation. The reliability of the correlation coefficient is evaluated with the confidence interval^55^. There are one-sided and two-sided significance tests. In the output Pearson value table, a single star at the upper right of the correlation coefficient means that it meets the one-sided test, and a double star means that it meets the two-sided test. If there is no star in the correlation coefficient of the corresponding index in the numerical table, it means that the two variables are not correlated or the correlation is not significant.

## Supplementary Information

Supplementary Information.

## Data Availability

The regional statistics data that support the findings of this study can be found in the supplementary document. The grid data sources of surface water, climate, and groundwater data can be downloaded from the original global product data set sharing website.

## Code availability

The code and module used in the study to extract the data of the study area from the global surface water and groundwater product data set are available on request from the corresponding author.
